# Evaluation of the Effect of Two Systemic Doses of HESA-A on Prevention of Induced Tongue Neoplasm in Rats

**DOI:** 10.5681/joddd.2013.035

**Published:** 2013-12-18

**Authors:** Masoumeh Mehdipour, Ali Taghavi Zenouz, Mehran Mesgari Abbasi, Daryoush Mohajeri, Hossein Damghani, Sanaz Helli, Bita Abdollahi

**Affiliations:** ^1^Associate Professor, Department of Oral Medicine, Faculty of Dentistry, Shahid Beheshti University of Medical Sciences, Tehran, Iran; ^2^Associate Professor, Department of Oral Medicine, Faculty of Dentistry, Tabriz University of Medical Sciences, Tabriz, Iran; ^3^DVM (Doctor of Veterinary Medicine), Drug Applied Research Center, Tabriz University of Medical Sciences, Tabriz, Iran; ^4^Associate Professor, Department of Pathobiology, Faculty of Veterinary Medicine, Tabriz Branch, Islamic Azad University, Tabriz, Iran; ^5^Post-graduate Student, Department of Oral Pathology, Faculty of Dentistry, Tabriz University of Medical Sciences, Tabriz, Iran; ^6^MScD in Oral Medicine, Dental and Periodontal Research Center, Tabriz University of Medical Sciences, Tabriz, Iran; ^7^MSc Histology and Embryology,Drug Applied Research Center, Tabriz, Iran

**Keywords:** 4-nitroquinoline-1-oxide, HESA-A, rat, tongue neoplasm

## Abstract

***Background and aims. ***The aim of the present study was to compare the inhibitory effects of two systemic doses of HESA-A on prevention of 4-NQO-induced tongue neoplasms in rats. This study evaluated weight and histopathological changes.

***Materials and methods.*** Forty-eight male Sprague Dawley rats were divided into four groups of A, B, C and D of each 12 rats. The rats in groups B to D received 30 ppm of 4-Nitroquinoline-1-oxide (4-NQO) in drinking water for 12 weeks.  When feeding with 4-NQO was initiated, the rats in groups B and C received HESA-A at doses of 250 and 500 mg/kg, respectively, 3 times a week. Body weights were measured three times a week. At the end, the rats were euthanized and the tongue was removed. Histological evaluations for carcinogenesis were carried out under a light microscope.

*** Results.*** The mean body weights of rats in groups B, C and D were significantly lower than that in group A (P < 0.05). There were no significant differences in weight changes between groups B, C and D. In the present study, after 12 weeks of treatment, Tongue specimens in groups B and C did not exhibit severe dysplastic changes; however, concurrent hyperplasia, without atypia and mild-to-moderate dysplastic changes were detected. These changes were significantly less than those in group D, with significant differences between group D and groups A, B and C (P<0.001, P<0.01 and P<0.05, respectively).

***Conclusion. ***HESA-A has dose-dependent inhibitory effects on the development of neoplasms of the tongue.

## Introduction

Oral cavity cancers are rather common and rank eleventh among other cancers all over the world.^1^ The incidence of oral cavity cancers, the major location of which is the tongue, has considerable variations in different parts of the world. The highest morbidity and mortality rates are seen in southern Asia due to the habit of chewing betel quid and tobacco.^2^ Based on reports by the World Health Organization tobacco and alcohol account for approximately 75% of all the oral cavity cancers all over the world.^3^

Prevention of oral cavity cancers has always been a major challenge for researchers because the oral cavity accessibility and the presence of clearly visible precancerous lesions (such as leukoplakias) make it easy to evaluate progression of disease.^4^ Due to the high mortality rate of oral cavity cancers (a five-year survival rate of approximately 60%)and the deleterious effect of surgical treatment modalities on the quality of life in a large number of patients with oral cavity cancers, prevention of oral cancers by chemo-preventive agents might prove efficacious.^5^ Researchers also continue to develop new chemotherapy drugs that might be more effective against advanced oral and oropharyngeal cancers. Newer approaches to treating head and neck cancers are New radiotherapy methods, Targeted therapy(such as growth factors and growth factor receptors), Gene therapy, Biological therapies( treatments made from naturally occurring body substances ) and Chemoprevention(e.g. certain extracts of black raspberries, green tea extracts and resveratrol).

HESA-A is an Iranian new immunomodulating medication with natural biological compounds, which has been patented in Iran for its biological properties. It has recently been permitted to be mass-produced by the Islamic Republic of Iran’s Ministry of Health and Medical Education (registration number: D-5-6638; dated on 14/06/2005). It is a mixture of herbal-marine substances and includes Penaeus latisculatus (king prawn), Carumcarvi and Apiumgraveolens with antineoplastic properties.^6^ The exact mechanism of HESA-A action on tumor cells is yet to be understood; however, it appears to exert several pharmacological effects. Analysis of the chemical composition of HESA-A has shown that it is composed of 50% inorganic substance, 45% organic substance (aminoenthraquinone) and 5% water. The inorganic component consists of calcium carbonate, magnesium phosphate and sulfate, potassium and sodium and elements such as aluminum, cobalt, potassium, chrome, iron, zinc, bromine and strontium at high concentrations.^7^ The substance is ground into a fine powder and pressed with wax C to prepare tablets for x-ray assay. A Phillips XRF 2404 x-ray fluorescent spectrometry (Tarbiat Modarres University) has been used for this assay.^8^

In toxicological studies, HESA-A caused no biochemical, hematological or histopathological signs of toxicity and a dose of 5000 mg/kg administered orally was estimated to be the no-observed-adverse- effect level (NOAEL) in mice and rats.^8^

In addition, a study on rabbits has shown hepato-protective functions of two doses of HESA-A (125 and 250 mg/kg) against hepatotoxicity induced by thioacetamide.^9^

HESA-A has a dose-dependent selective inhibitory effect on the growth of neoplastic cells, possibly through interaction with cellular DNA, which is a source of concern. In addition, the apoptotic effects of HESA-A may play an important role in its antineoplastic effects.^10,11^

The compound also exhibited dose-dependent selective inhibitory effects, similar to those of doxorubicin, on the growth of malignant human cell lines, without significantly affecting normal cells.^12^

Administration of 4-nitroquinoline-1-oxide (4NQO) in drinking water produces some precancerous and malignant lesions on the dorsal aspect of the rat tongue.^13^ A number of synthetic and naturally occurring agents have been experimented for their chemo-preventative effects on oral cancers induced by 4NQO.^14^

The effect of HESA-A on oral cancers has not been evaluated to date and this is the first study in this regard. The aim of this study was to investigate and compare the inhibitory effects of HESA-A on 4NQO-induced tongue neoplasms in rats. This study had two main aims: weight changes and histopathologic changes.

## Materials and Methods

### Animals

Forty-eight adult male Sprague Dawley rats (3‒3.5 month-old) with an average weight of 220 g were obtained from the Animal Lab of Tabriz University of Medical Sciences. The animals were quarantined and acclimatized to laboratory conditions for 2 weeks. During the study, each rat was housed in a metal cage, with hardwood chips for bedding in an air-conditioned room under 12-h light/12-h dark cycles at a temperature of 22±2°C.

### Chemicals 

4-NQO was purchased from Sigma Inc. (Germany). 4-NQO solution (30 ppm) was prepared twice weekly by dissolving the carcinogenic agent in distilled water and was given in light-opaque covered bottles. The rats had access to drinking water ad libitum (this phrase denotes providing an animal free access to feed or water thereby allowing the animal to self-regulate intake according to its biological needs), during the experiment. HESA-A powder was dissolved for an hour in normal saline solution acidified with HCl. The resultant solution was treated with NaOH to reach a final pH of 7.4. The solution was filtered and administered to rats by gavage three times a week at doses of 250 and 500 mg/kg. Normal saline and 4NQO (30 ppm) were used as negative and positive controls, respectively.

###  Experimental Procedure 

Forty-eight 12-week-old male Sprague Dawley rats, were randomly divided into four groups of A, B, C, and D (n=12). Group A served as the control and was fed on basic diet and tap water without 4-NQO. The rats in groups B to D received 30 ppm of 4-NQO in drinking water for 12 weeks. When feeding with 4-NQO was initiated the rats in groups B and C received HESA-A at doses of 500 and 250 mg/kg, respectively, 3 times a week. Body weights were measured three times a week. Some rats died during the experiment. All the procedures complied with animal care protocol and the regulations observed by Tabriz University of Medical Sciences Animal Care and Use Committee.

At the end of the experiment, the rats were euthanized and the tongue was removed. Tissue specimens collected from tongues were fixed in 10% buffered formalin and embedded in paraffin; microscopic sections of the tongue measuring 5 μm in thickness were prepared by hematoxylin-eosin staining method. Histological evaluations for carcinogenesis were carried out. Tongue epithelial lesions were diagnosed based on criteria introduced by Baonczy and Csiba^15^and Kramer et al.^16^

### Evaluation of Sections 

The specimens underwent a blind qualification process by one histologist and one pathologist. Histological slides were evaluated under a light microscope (Olympus BX40, Tokyo, Japan). In H&E-stained slides, thickness of tongue epithelia was measured at five different fields using the software Motic-imageplus II and a mean was recorded as the thickness of the epithelial layer. Pathological changes including dysplasia, hyperplasia, hyperkeratosis, parakeratosis, tumor-like cells and pearl bodies in the epithelia were graded according to WHO criteria as intact, mild, moderate and severe.^15^

### Statistical Analysis 

Fisher's exact or chi-squared tests were used for statistical analysis on the incidence of lesions and data on body weight were compared by ANOVA. Statistical significance was defined at P<0.05.

## Results

**Figure 1.**
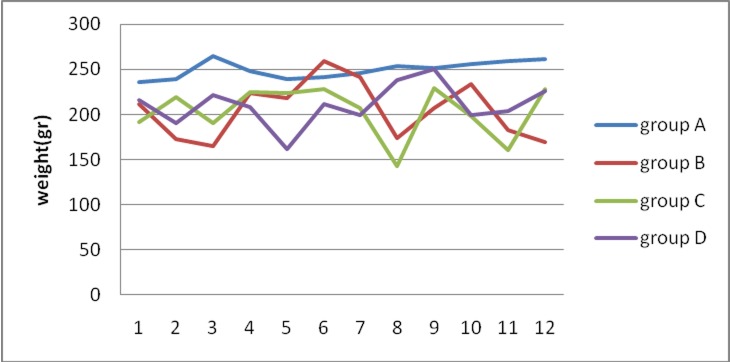
Weight changes in the studied groups.

In the present study 8 of rat died during experiment. 3 of group B, 2 of group C and 3 of group D. The mean body weights at the end of the study are presented in Table 1 and the weight changes are shown in Figure 1. The mean body weights of rats in groups B, C and D were significantly lower than that in group A (P<0.05). There were no significant differences in weight changes between groups B, C and D.

**Table 1:** Body weight in each group

Group	Treatment	No. of rats examined	Weight(gr) Mean
A	Control	12	244.48±10.93^a^
B	4-NQO+500 mg/kg HESA-A	9	210.84±31.73^b^
C	4-NQO+250 mg/kg HESA-A	10	205.053±28.63^b^
D	NQO alone	9	203.77±23.25^b^
Different superscripts show significant difference from group A by ANOVA test (followed by post hoc Duncan test) (P<0.05).

In the present study, after 12 weeks of treatment, hyperplasia and three types of dysplasia (mild, moderate and severe), considered precancerous lesions in the oral cavity, were present in the tongue of rats in groups B to D, but not in rats in group A. Almost all the rats in group D exhibited hyperplasia and all types of dysplasia. Tongue specimens in groups B and C did not exhibit severe dysplastic changes; however, concurrent hyperplasia, without atypia, and mild-to-moderate dysplastic changes were detected. These changes were significantly less than those in group D, with significant differences between group D and groups A, B and C (P<0.001, P<0.01 and P<0.05, respectively). The frequencies of hyperplasia and dysplasia in group B were significantly lower than those in group C (P<0.05). The incidence of moderate dysplasia in rats in group B was significantly less than that in group C (P<0.05). However, only one rat from group B (given HESA-A at a dose of 500 mg/kg during 4-NQO administration) had moderate dysplastic changes. The incidence rates of such lesions are listed in Tables 2 and 3.

**Table 2:** Incidence of tongue preneoplastic changes of rats given 4-NQO together with HESA-A

Group	Treatment	No. of rats examined	No. of rats (%)
			Normal	Hyperplasia	Dysplasia
A	Control	12	12/12^a^	0/12^a^	0/12^a^
B	4-NQO+500 mg/kg HESA-A	9	3/9^b^(33)	6/9^b^(67)	3/9^b^(33)
C	4-NQO+250 mg/kg HESA-A	10	2/10^c^(20)	8/10^c^(80)	5/10^c^(50)
D	NQO alone	9	0/9	9/9(100)	8/9(88)
Different superscripts show significant differences from group D by Fisher’s exact probability test (^a^ P<0.001, ^b^ P<0.01 and ^c^ P<0.05).

**Table 3:** Incidence of tongue dysplasia in rats of each group

Group	Treatment	No. of rats with dysplasia	No. of rats (%)
			Mild dysplasia	Moderate dysplasia	Severe dysplasia
A	Control	0/12	0/12^a^	0/12^a^	0/12^a^
B	4-NQO+500 mg/kg HESA-A	3/9	2/9(22)	1/9b(11)	0/9^b^
C	4-NQO+250 mg/kg HESA-A	5/10	3/10^b^(30)	2/10^c^(20)	0/10^b^
D	NQO alone	8/9	2/9(22)	3/9(33)	3/9(33)
Different superscripts show significant differences from group D by Fisher’s exact probability test (^a^ P<0.001, ^b^ P<0.01 and ^c^ P<0.05).

**Figure 2.**
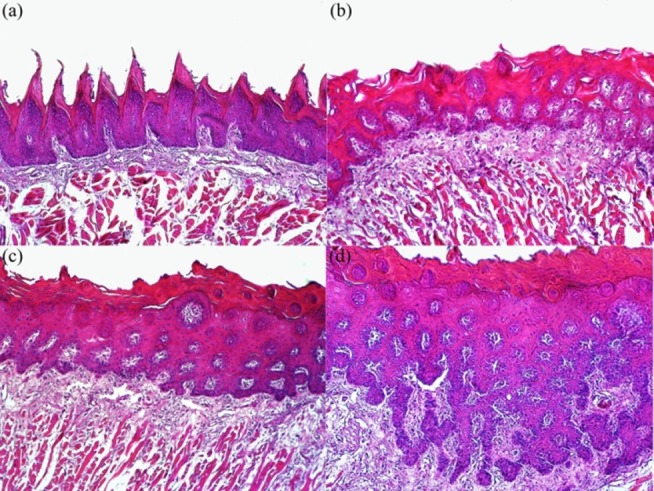
Histologic appearance of tongue epithelium of rats 12 weeks after starting the experiment (H&E staining, original magnification ×40). (a: control group) Normal tongue with no histological changes. (b: 4-NQO+500 mg/kg of HESA-A) Mild hyperplasia and hyperkeratosis. (c: 4-NQO+250 mg/kg of HESA-A) Moderate hyperplasia and hyperkeratosis. (d: 4-NQO) Severe hyperplasia and hyperkeratosis.

**Figure 3.**
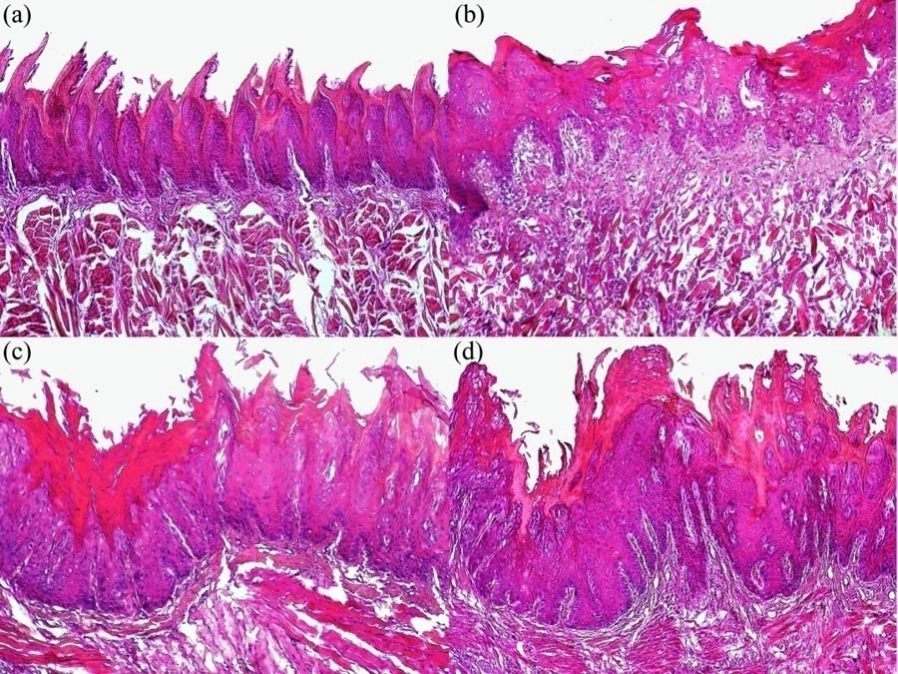
Histologic appearance of tongue epithelium of rats 12 weeks after starting the experiment (H&E staining, original magnification ×40). (a: control group) Normal tongue with no histological changes. (b: 4-NQO+500 mg/kg of HESA-A) Mild dysplasia and hyperkeratosis. (c: 4-NQO+250 mg/kg of HESA-A) Moderate dysplasia and hyperkeratosis. (d: 4-NQO) Severe dysplasia and hyperkeratosis.

Microscopically, no histological changes in tongue basal epithelia were observed in the control group (Figures 2,3). Mild histological changes, including hyperplasia and hyperkeratosis with thickened spinous cell layer (Figure 2), were observed after 12 weeks of treatment in tongue basal epithelia in group B (4-NQO + 500 mg/kg of HESA-A). In this group, tongue basal epithelial dysplasia was mainly found in mild form (3). In addition to moderate hyperplasia and hyperkeratosis throughout the whole thickness of tongue epithelia (Figure 2), moderate dysplasia of tongue basal epithelia was also found in group C (4-NQO + 250 mg/kg HESA-A), (Figure 3). Despite severe epithelial hyperplasia of tongue (Figure 2), evidence of severe dysplasia in tongue basal epithelia, as a precancerous change, was identified in group D (4-NQO) (Figure 3).

## Discussion

Cancer is the most common cause of mortality in many countries.^1^ In most malignant conditions there is no effective treatment available. At present, after a diagnosis of a malignancy, selection of a treatment modality depends on the type and progress of the condition. Chemotherapy is mainly used for advanced and metastatic cancers.^3^In stage I and II, chemotherapy has no main role as an anti-neoplastic agent, while it is as an adjunctive medicine in the treatment of stage III and IV. Chemotherapy agents are generally divided into chemical and synthetic groups. Despite recent advances in pharmacology and pharmaceuticals 25% of all the medicines prescribed in medical sciences are derivatives of natural products. In the case of antineoplastic agents this rate is more than 80%.^5^

Attempts now are under way to preserve or improve the quality of life of patients with the help of traditional and adjunctive medicine so that the potentials of these medical disciplines can be more properly used in this field. However, it does not mean that they should replace standard cancer treatments.^6^ Traditional methods and medications, along with herbal medicines, are considered adjunctive therapy. In other words, the attitude of traditional and supplementary medicine toward oncology is to add adjunctive treatment modalities to aggressive and destructive cancer treatments. In this context, several research studies and clinical trials have shown the efficacy of traditional and adjunctive medicine in the treatment of cancer and improvement of the quality of life of cancer patients. HESA-A is a natural product with a herbal/marine origin; its antineoplastic effects have been tested in vivo^17^ and in vitro^12^and it has selective effects on tumor cells. The cytotoxicity of HESA-A has been tested, too, and fortunately, it has not exhibited any cytotoxic effects on normal cells.^9^ It contains mineral agents and rare elements such as selenium, strontium, cobalt, chromium, zinc and molybdenum. It exerts its antioxidative and anti-inflammatory effects through induction of apoptosis. As an example, selenium is an antioxidative agent used in cancer treatment; it enters cancerous cells and destroys these cells by an alkalinization process. Strontium is an essential rare element, which promotes the growth of normal cells.^18^ It appears that this medicine exerts it antineoplastic activity via the elements it contains.

In the present study, the effect of HESA-A on prevention of induced neoplasms in rat tongue was evaluated by monitoring weight changes and histopathologic changes. HESA-A is produced in Iran. The results of this study showed a significant weight loss in the groups receiving the medication compared to the negative control group at the end of the study. However, there were no significant differences in weight loss between the groups receiving the medication (B, C and D). In other words, groups B, C and D, which had received 4NQO alone or in combination with different doses of HESA-A, had experienced weight loss, which might be attributed to the use of these medications. On the other hand, there were no significant differences in weight loss between the three groups, i.e. use of HESA-A at 250- and 500-mg/kg doses did not result in weight loss, consistent with the results of a study by Hajhashemi in 2001, who reported no changes in weight after administration of HESA-A.^9^ The results of the present study are not consistent with those reported by Balali et al in 2006. In that study, toxic doses of HESA-A (higher than 5000 mg/kg) resulted in weight loss in the animals under study.^19^ On the other hand, Sohrabi et al reported in 2009 that the carcinogenic medicine 4NQO results in weight loss and pathologic changes in the composition and size of cells.^20^ Therefore, it can be concluded in general that weight loss is mainly induced by 4NQO rather than by HESA-A. It should be pointed out that evaluation of the side effects of 4NQO and HESA-A was not one of the aims of the present study and more precise evaluation of the reasons behind weight loss is necessary.

The chief aim of the present study was to evaluate dysplastic changes in the tongue in groups under study and comparison of the severity of dysplasia induced.

The results showed no dysplasia in the negative control group. However, there were different degrees of dysplasia in the three groups receiving the medicine, i.e. the carcinogenic agent had induced its neoplastic effects in the tongue of the rats. On the other hand, the severity of dysplasia was different in the three groups. In the group which had received only the carcinogenic 4NQO medication, severe dysplasia (the stage before carcinoma in situ and malignancy) was observed. However, in the two groups receiving 4NQO along with HESA-A, none of the rats exhibited severe dysplasia. In addition, in the group receiving a higher dose of HESA-A, moderate dysplasia was observed, which was less than that in other groups. Generally, the severity of dysplasia decreased with the administration of HESA-A, with a decrease in its intensity as HESA-A dose increased; the difference was statistically significant, i.e. HESA-A exhibited an inhibitory effect on the induction of dysplasia, which was dose-dependent: the inhibitory effect increased with an increase in the dose of HESA-A.

In another in vivo study on rabbits in 2001, daily doses of 350 mg/kg of HESA-A caused shrinkage of osteosarcoma tumors and resulted in complete disappearance of tumor in 10 weeks. Rabbits in the study group survived, whereas those in the control group expired during the same period. Pathological studies confirmed the effects of HESA-A on the shrinkage of induced osteosarcoma.^17^

In addition, a study by Moallem et al in 2009 showed that HESA-A can retard the fetal growth in mice. They reported that administration of the medicine at a dose of 400 mg/kg in pregnant mice can induce clear structural anomalies in fetus and the effect is completely dose-dependent,^21^ indicating that administration of this medicine in pregnant mice should be carried out with more caution.

In a double-blind clinical trial in 2005, 24 patients suffering from breast cancer with choroidal metastasis were treated with HESA-A at a dose of 50 mg/kg/day and the findings indicated significant improvement of vision and pain toleration in patients compared with the control group.^22^

In a study in 2009, 30 patients with hepatic metastatic cancers underwent treatment with 50 mg/kg of HESA-A daily for 2 months, which resulted in a significant increase in appetite and a significant decrease in pain severity. There were generally positive effects, including an increase in efficacy, improvements in life quality and a decrease in complications.^11^

In another study in 2010, 50 patients with advanced colon cancer and with hepatic metastasis were evaluated for 6 months. A definitive increase in quality of life was observed based on Karnovski criteria, without any side effects. The positive results included an increase in apatite, an improvement in performance, a feeling of well-being and a decrease in pain severity. In addition, the survival of patients until the end of the study, despite a predication of mean survival rate of 1 month, was noteworthy.^23^ Ahmadi carried out a study in 2010 on patients with metastatic cancers, who had received HESA-A, and reported that this medicine can improve the quality of life and survival rate in such patients.^24^ It is obvious that more animal studies are necessary with different doses in order to determine the optimal dose to prevent oral cancers, paving the way for human studies.

## Conclusion

Based on the results of the present animal study, HESA-A has dose-dependent inhibitory effects on the development of neoplasms of the tongue. However, there were no significant differences in weight loss between the groups receiving the medication.

###  Acknowledgments

This article is based on the dataset from a MScD thesis in Oral Medicine, entitled “Evaluation of the Effect of Two Systemic Doses of Hesa-A on Prevention of Induced Tongue Neoplasm in Rats” (reference number T/133) registered in Tabriz University of Medical Sciences. The authors would like to thank Seemorgh Hekmat Research Institute for providing the medications, the Laboratory Animal Research Center at Applied Drugs Research Center, Tabriz University of Medical Sciences and Research Vice Dean of Faculty of Dentistry for their assistance in carrying out the experiments.
